# Quality of life, level of functioning, and its relationship with mental and physical disorders in the elderly: results from the MentDis_ICF65+ study

**DOI:** 10.1186/s12955-020-01310-6

**Published:** 2020-03-06

**Authors:** Luigi Grassi, Rosangela Caruso, Chiara Da Ronch, Martin Härter, Holger Schulz, Jana Volkert, Maria Dehoust, Susanne Sehner, Anna Suling, Karl Wegscheider, Berta Ausín, Alessandra Canuto, Manuel Muñoz, Mike J. Crawford, Yael Hershkovitz, Alan Quirk, Ora Rotenstein, Ana Belén Santos-Olmo, Arieh Shalev, Jens Strehle, Kerstin Weber, Hans-Ulrich Wittchen, Sylke Andreas, Martino Belvederi Murri, Luigi Zerbinati, Maria Giulia Nanni

**Affiliations:** 1 Institute of Psychiatry, Department of Biomedical and Specialty Surgical Sciences, University of Ferrara, Via Fossato di Mortara 64a -, 44121 Ferrara, Italy; 2 Department of Medical Psychology, University Medical Centre Hamburg-Eppendorf, Martinistr. 52, Building W 26, D-20246 Hamburg, Germany; 3 Department of Psychosocial Prevention, University of Heidelberg, Bergheimer Str. 54, 69115 Heidelberg, Germany; 4 Institute of Medical Biometry and Epidemiology, University Medical Centre Hamburg-Eppendorf, Martinistr, 52, Building W 34, D-20246 Hamburg, Germany; 5 School of Psychology, University Complutense of Madrid, Campus de Somosaguas s/n, 28223 Madrid, Spain; 6 Nant Foundation, East Vaud Psychiatric Institute, Route de Nant, 1804 Corsier-sur-Vevey, Switzerland; 7 Royal College of Psychiatrists, Mansell Street 21, E18AA, London, UK; 8 Hadassah University Medical Centre, P.O.B 12000, 91120 Jerusalem, Israel; 9 Department of Psychiatry, Langone Medical Center, New York, NY USA; 10 Institute of Clinical Psychology and Psychotherapy, Technische Universitaet Dresden, Chemnitzer Straße 46, 01187 Dresden, Germany; 11 Curabilis, Medical Direction, University Hospitals of Geneva, Chemin de Champ-Dollon 20, 1241 Puplinge, Switzerland; 12 Institute for Psychology, Alpen-Adria Universität Klagenfurt, A-9020 Klagenfurt, Austria

**Keywords:** Quality of life, Functioning, Psychiatric morbidity, Physicial morbidity, Elderly

## Abstract

**Background:**

An ageing population worldwide needs to investigate quality of life (QoL) and level of functioning (LoF) in the elderly and its associated variables. We aimed to study the relationship between Quality of Life (QoL) and Level of Functioning (LoF) in an elderly population in Europe.

**Method:**

As part of the Ment_Dis65+ European Project, 3142 community-dwelling adults aged 65–84 years in six countries were assessed by using the adaptation for the elderly of the Composite International Diagnostic Interview (CIDI65+) to provide psychiatric diagnosis according to the International Classification of Diseases (10th edition) (ICD-10 Classification of Mental and Behavioural Disorders). Socio-demographic and clinical interviews, and two self-report tools, the World Health Organization QoL assessment (WHO QoL BREF), to assess QoL, and the WHO Disability Assessment Schedule -II (WHODAS-II), to assess LoF, were also administered.

**Results:**

Most subjects reported good levels of QoL (56.6%) and self-rated health (62%), with no or mild disability (58.8%). There was a linear decrease of the QoL and the LoF by increase of age. Elderly with ICD-10 mental disorder (e.g. somatoform, affective and anxiety disorders) had poorer QoL and lower LoF. There were a number of predictors of lower levels of QoL and disability, including both socio-demographic variables (e.g. male gender, increase in age, poor financial situation, retirement, reduced number of close significant others), ICD-10 psychiatric diagnosis (mainly anxiety, somatoform disorders) and presence of medical disorders (mainly heart and respiratory diseases).

**Conclusions:**

The study indicates that QoL and LoF were quite acceptable in European elderly people. A series of variables, including psychiatric and somatic disorders, as well as socio-demographic factor influenced in a negative way both QoL and LoF. More specific links between mental health, social and health services dedicated to this segment of the population, should be implemented in order to provide better care for elderly people with conditions impacting their QoL and functioning.

## Background

Because of the increasing number of people aged 65 worldwide, especially in Europe, where 30% of the population will be over 65 by 2050, [1] mental health in the elderly and its relationship with quality of life (QoL) and levels of functioning has become increasingly important [2–4].

With respect to this, in the English Longitudinal Study of Ageing, poor QoL in older adults was shown to be related to the presence of depression and problems in maintenance of physical function, difficult neighborhood standards, family relationships and financial circumstances [5, 6]. Recently, a reciprocal interrelationship between mental health disorders and chronic diseases was found in the elderly, with pre-existing mental disorders contributing to the development of chronic disease over time, and pre-existing chronic disease(s) contributing to the development of mental health disorders over time [7]. Also, disability and poor level of functioning has been found to be an independent risk factor for depression, [8] and as a key dimension associated with impairment of quality of life, [9] which, in turn, is associated with mental disorders [10]. Similar results, confirming these data, have been reported in other studies carried out in Spain, [11, 12] and Asia [13, 14] indicating that mental disorders, especially anxiety, depression and adjustment disorders, had a higher impact of on health related QoL and LoF, than chronic physical conditions.

Most studies however have been conducted in a single center or nation, making it difficult to compare by using common instruments, possible differences between countries and suggesting the need for more international studies [15]. The only multicenter study available in Europe is the European Study of the Epidemiology of Mental Disorders - ESEMeD project) which, however, was not specifcially devised for the elderly population. When looking at the part of the sample aged over 75, 68.8% resulted to have problems in one or more dimensions of a standardized measures for health outcome [16]. Mental disorders, mainly anxiety disorders, followed by mood disorders, and alcohol disorders were related to disability in all domains of functioning, to the same degree or higher than physical disorders, [17] with lowest levels of QoL in the Netherlands and highest in Italy [18].

The MentDis_ICF65+ multicentre research project^,^ funded by the European 7th Framework Research Program, had the main aim to examine the prevalence, 1-year incidence and symptom severity of mental disorders in the elderly, in relationship to impairment, functioning (ICF) and service utilization, [19] to allow for comparability of epidemiological data across cultures. The aims of the study presented here were to explore the characteristics of QoL and LoF among the elderly in European countries and the association of mental disorders, as examined by using used an adapted, age-appropriate version of the CIDI (CIDI65+), and other medical and socio-demographic variables with QoL and LoF.

## Methods

The MentDis_ICF65+ study is a multicenter survey conducted in six countries (Germany, Italy, England, Spain, Switzerland, and Israel). Each study center defined a catchment community area representative of the region’s population with regard to social class (equal inclusion of working, middle, and upper social class populations), specific living conditions of our age cohort (e.g., no exclusion of nursing homes), and population structure (urban/rural), as has been described in detail elsewhere. A random sample of 500 older adults for each country (drawn from the population registries in Hamburg and Ferrara and from postal addresses of market research units in Madrid, Geneva, London/Canterbury and Jerusalem, stratified by gender and age (65–69, 70–74, 75–79, 80–84 years), from all those living in selected catchment areas was recruited. Inclusion criteria for participants were the ability to provide informed consent, living in the predefined catchment area at the beginning of the study and being between 65 and 84 years old. Exclusion criteria were severe cognitive impairment (Mini-Mental State Examination Mini cut-off score > 18) [20] and insufficient level of the corresponding language. A harmonized procedure in contacting subjects and conducting the survey was realized, including initial contact by phone and mail, standardized interviewer training, implementation of a standardized study protocol for all centers, and using stringent, high quality data control procedures. The recruitment and consent procedure was approved by research ethics committees in all participating countries. A detailed description of the methodology has been provided elsewhere, including data regarding the analysis of representativeness, that showed only significant differences with a small effect size for some sociodemographic characteristics between the MentDis_ICF65+ study sample and the population of the respective catchment area and the country of each study center [19, 21].

### Measures

Data were collected using computer-assisted personal interviewing (CAPI). Interviewers received extensive training prior to the start of the study, and the ability to clinically conduct a psychiatric interview was assessed in a training workshop conducted in the PI Hamburg site and during the pilot study [19]. Their activity was monitored and supervised continuously during the course of data collection by the PI of each participating center [22]. The interviews took place in the participants’ homes. The Composite International Diagnostic Interview for the Elderly (CIDI65+) was developed by the study group and has been adapted to the particular social, cognitive and psychological abilities and needs of the elderly. The interview covers several mental health problems such as anxiety disorders, affective disorders and substance abuse. Preliminary evidence of satisfactory test–retest reliability and the feasibility of this extended and modified CIDI approach is for this particular study has been reported [22]. Considering that previous investigation showed that the strongest significant impact on QoL and disability was determined mainly by anxiety and mood disorders, [18] for the aims of the present study, besides the global analysis of all mental disorders prevalence, we particularly focused attention on anxiety, depression and somatoform disorders in their possible association with QoL and LoF. During the interview, participants were also asked for the existence of any medical condition through a specific checklist inquiring for physical disorders (e.g. heart disease, cancer, musculoskeletal disease, endocrinological disorders) also to examine the influence of physical diseases to somatoform disorders [23].

Two self-rated questionnaires to assess levels of quality of life and functioning were used. QoL was evaluated by the WHO Quality of Life in its brief version (WHO-Qol-BREF) [24] which is a widely used instrument with good psychometric properties and that can be successfully administered in older people [25, 26]. It examines an individual’s perceptions in the context of their culture and value systems, and their personal goals, standards and concerns by inquiring ‘how much’, ‘how completely’, how often’, ‘how good’ or ‘how satisfied’ the respondent felt in the last 2 weeks. The questionnaire measures four dimensions, including physical and psychological well-being, environmental factors, and social support, that together yields a Global score of QoL, with a score range of 0 to 100 (higher scores corresponding to better quality of life). Two additional questions (“Overall, how would you rate your quality of life?” and “How satisfied are you with your health?”) also give general information (on a 0–100 VAS) about the personal view of the individual QoL. In the present study a shortened version of the questionnaire was used, whereby satisfying correlation coefficients of *r* = 0.78 to 0.91 could be found between the reduced scale and the original scale in the pilot sample of the MentDis_ICF65+ study [19].

LoF was measured by a 12-item self-administered version of the World Health Organization Disability Assessment Schedule II (WHODAS-II) [27]. Respondents were asked to state the level of difficulty experienced taking into consideration how they usually do the activity, including the use of any assistive devices and/or help from another person. For each item, individuals had to estimate the magnitude of the disability during the previous 30 days using a five-point scale (none = 1, mild = 2, moderate = 3, severe = 4, extreme/cannot do = 5). Functional impairment of daily activities (disability) had six domains, namely understanding and communicating (cognitive domain); moving and getting around (mobility domain); personal hygiene, dressing, eating and ability to live alone (self-care domain); getting along with others and interaction with other individuals (social domain); carrying out work or life activities (household domain); and participation in society (society domain). Higher scores in WHODAS-II represent lower levels of functioning.

A further item (“How do you rate your overall health in the past 30 days”) was added, with a score of 1 corresponding to “very good” and 5 to “very bad” (Self-Health Rated-SHR).

### Statistical analysis

Sample characteristics are given as mean and standard deviation. All survey analyses were weighted (regarding the number of inhabitants) and take into account the stratification (four strata by gender and two age groups: 65–74 and older than 74 years). Linear regressions were used to analyse the effect of four age groups (65–69, 70–74, 75–79, > 80), gender, study centre, socio-demographic factors, mental and physical disorders on the WHODAS-II and WHO-Qol-BREF as well as their subscales. Moreover the effect of WHO-QoL-BREF subscales and self-rated health (SRH) on the WHODAS-II was estimated using linear regression, adjusted for age, gender and centre. Effects are presented as model coefficients with 95%-confidence intervals (CI). In all models tentatively, an interaction term of sex and age was added and kept in the model if significant. A two-tailed *p*-value < 0.05 was considered statistically significant. All analyses were conducted using Stata 13.1 (STATA Corporation, College Station, Texas US).

## Results

### Sample characteristics

As detailed elsewhere [19], each participating site interviewed a heterogeneous sample, equally distributed across two age groups (65–74 young-old, and 75-85 years old-old). The final sample consisted of 3142 people (mean age 73.7 ± 5.6 years), with an equal number of male (*n* = 1550, 49.3%) and female (*n* = 1592, 50.7%). Most were married (61.0%) and retired (84.6%). Education was 10.3 years on average (SD = 3.2, range: 8–13 years) with most subjects graduating from the last school they attended (76.1%). A total of 55% estimated their financial situation as good to very good, 36.6% thought they had just enough, and 8.2% estimated it to be poor to very poor (see [28] for the details about sample characteristics].

### QoL and LoF characteristics of the sample

Table 1 provides the descriptive data (mean scores for each item) for the WHO-QoL-BREF the WHODAS-II and the SHR by gender, age and center.

**Table 1:** Descriptive data of WHO QoL-BREF and Level of Functioning (WHODAS II) measures in *N* = 3142 European community-dwelling elderly

	Overall	Gender		Age				Study centre					
		Male	Female	65–69	70–74	75–79	80–84	Hamburg	London	Madrid	Ferrara	Geneva	Jerusalem
**WHO QoL-BREF (Range all scores: 0–100)**													
Global	67.2 ± 18.2	68.5 ± 17.9	66.0 ± 18.3^a^	68.6 ± 18.4	66.5 ± 19.0	68.2 ± 17.6	64.1 ± 16.9^a^	68.2 ± 18.7	70.6 ± 18.1	64.6 ± 18.3	63.5 ± 16.4	77.5 ± 15.6	63.5 ± 19.8^a^
Environment	72.1 ± 15.9	73.8 ± 14.9	70.8 ± 16.6^b^	72.8 ± 16.2	71.9 ± 16.5	72.8 ± 15.1	70.2 ± 15.3^b^	71.8 ± 15.4	76.0 ± 15.6	71.1 ± 13.4	68.7 ± 16.5	87.9 ± 13.3	62.9 ± 22.2^a^
Social	77.6 ± 17.3	77.2 ± 16.7	77.9 ± 17.8	78.3 ± 17.6	76.6 ± 17.4	78.7 ± 16.9	76.3 ± 16.9	78.7 ± 16.1	82.6 ± 18.0	76.3 ± 16.3	72.4 ± 17.2	82.3 ± 15.2	71.6 ± 23.3^a^
Physical	70.2 ± 19.8	71.5 ± 19.4	69.1 ± 20.0^a^	74.1 ± 19.2	71.3 ± 19.4	68.9 ± 19.1	62.7 ± 20.4^a^	72.6 ± 19.4	68.9 ± 20.7	68.3 ± 18.5	69.1 ± 19.7	73.0 ± 19.9	66.8 ± 24.6^a^
**WHODAS II (Range total score: 12–60; Range subscales: 1–10)**													
Total Score	17.5 ± 6.7	16.6 ± 6.4	18.2 ± 6.9^a^	15.9 ± 5.8	17.1 ± 6.2	17.8 ± 6.5	20.9 ± 8.2^a^	17.5 ± 6.4	18.4 ± 7.3	16.9 ± 6.1	17.1 ± 7.0	15.9 ± 4.8	20.9 ± 8.8^a^
Mobility	3.9 ± 2.4	3.5 ± 2.1	4.2 ± 2.5^a^	3.2 ± 2.0	3.6 ± 2.1	4.1 ± 2.3	5.2 ± 2.7^a^	3.8 ± 2.3	4.3 ± 2.5	3.6 ± 2.2	3.8 ± 2.4	3.2 ± 2.0	4.5 ± 2.5^a^
Household	3.0 ± 1.7	2.8 ± 1.5	3.2 ± 1.8^a^	2.7 ± 1.5	2.9 ± 1.6	3.1 ± 1.7	3.7 ± 2.1^a^	3.1 ± 1.7	3.2 ± 1.7	2.8 ± 1.4	2.9 ± 1.8	2.7 ± 1.3	3.8 ± 2.1^a^
Cognitive	2.6 ± 1.2	2.5 ± 1.1	2.7 ± 1.2^b^	2.4 ± 1.0	2.6 ± 1.1	2.7 ± 1.2	2.9 ± 1.4^b^	2.6 ± 1.1	2.8 ± 1.3	2.7 ± 1.2	2.5 ± 1.1	2.5 ± 0.9	3.2 ± 1.8^a^
Social	2.4 ± 1.0	2.4 ± 1.0	2.3 ± 0.9	2.3 ± 0.8	2.4 ± 1.1	2.4 ± 1.0	2.4 ± 1.0	2.4 ± 1.0	2.4 ± 1.0	2.4 ± 1.0	2.3 ± 0.9	2.4 ± 0.8	2.8 ± 1.5
Self-care	2.4 ± 1.2	2.4 ± 1.2	2.4 ± 1.2	2.2 ± 0.9	2.3 ± 1.1	2.3 ± 1.1	2.9 ± 1.8^a^	2.3 ± 1.0	2.6 ± 1.4	2.3 ± 1.1	2.4 ± 1.4	2.2 ± 0.6	2.7 ± 1.7^a^
Society	3.2 ± 1.6	3.0 ± 1.5	3.4 ± 1.6^a^	3.0 ± 1.5	3.2 ± 1.5	3.2 ± 1.5	3.7 ± 1.9^a^	3.3 ± 1.5	3.2 ± 1.5	3.1 ± 1.5	3.2 ± 1.7	2.9 ± 1.3	3.8 ± 2.1^a^
**SHR (Range: 1–5)**													
Global	2.3 ± 0.8	2.2 ± 0.8	2.4 ± 0.8^a^	2.2 ± 0.8	2.3 ± 0.8	2.4 ± 0.8	2.6 ± 0.8^a^	2.3 ± 0.8	2.2 ± 0.9	2.4 ± 0.8	2.5 ± 0.8	2.0 ± 0.7	2.4 ± 0.9^a^

**Legend:** Table shows weighted means ± standard deviations. The effect of gender, age (and interaction of age and gender if significant) and study centre on the respective scale was tested within a multiple linear regression, adjusting for the survey structure of the data (see methods section). ^a^ indicates a significant main effect < 0.05; ^b^ indicates a significant interaction between age and sex.

WHODAS II = WHO Disability Assessment Schedule, WHO QoL-BREF = WHO Quality of Life BREF; SHR = Self-Health Rated (*How do you rate your overall health in the past 30 days?)*

**Table 2:** Adjusted mean scores for quality of life (WHO-QoL-BREF) and level of functioning (WHODAS-II)) in elderly with and without an ICD-10 diagnosis of mental disorders.

Scale	Any mental disorder			Major Depression			Anxiety Disorders			Somatoform Disorders		
	Yes	No	p	Yes	No	p	Yes	No	p	Yes	No	p
**WHO-QoLBref**												
Physical	64.7 [63.5;66.0]	73.2 [72.1;74.3]	***< 0.001***	66.1 [63.6;68.7]	70.7 [70.1;71.2]	***0.003***	63.3 [61.3;65.3]	71.6 [71.0;72.2]	***< 0.001***	55.6 [52.0;59.2]	70.8 [70.3;71.4]	***< 0.001***
Social	73.6 [71.7;75.4]	79.8 [78.9;80.6]	***< 0.001***	72.1 [69.2;75.0]	78.2 [76.9;79.5]	***0.002***	73.7 [71.4;76.1]	78.4 [77.4;79.3]	***< 0.001***	71.2 [67.6;74.9]	77.8 [76.8;78.9]	***< 0.001***
Environment	68.9 [67.1;70.6]	73.9 [72.8;75.0]	***< 0.001***	70.2 [68.5;71.8]	72.4 [71.5;73.2]	***0.013***	69.0 [66.7;71.2]	72.8 [71.8;73.7]	***0.006***	63.6 [59.1;68.1]	72.5 [71.7;73.3]	***< 0.001***
Global	61.7 [60.4;62.9]	70.1 [69.0;71.3]	***< 0.001***	63.6 [61.3;65.8]	67.6 [66.6;68.5]	***0.002***	61.7 [60.2;63.1]	68.3 [67.3;69.3]	***< 0.001***	52.7 [48.3;57.0]	67.8 [66.8;68.7]	***< 0.001***
**WHODAS II**												
Mobility	4.4 [4.2;4.7]	3.6 [3.4;3.7]	***< 0.001***	4.2 [3.8;4.6]	3.8 [3.7;3.9]	0.060	4.2 [3.9;4.5]	3.8 [3.7;3.9]	***0.026***	5.6 [5.1;6.1]	3.8 [3.7;3.9]	***< 0.001***
Household	3.5 [3.4;3.7]	2.7 [2.6;2.8]	***< 0.001***	3.5 [3.2;3.8]	3.0 [2.9;3.0]	***0.004***	3.4 [3.2;3.6]	2.9 [2.9;3.0]	***< 0.001***	4.3 [4.0;4.7]	3.0 [2.9;3.0]	***< 0.001***
Cognitive	2.9 [2.8;3.0]	2.5 [2.5;2.5]	***< 0.001***	2.9 [2.7;3.1]	2.6 [2.6;2.7]	***0.025***	2.9 [2.8;3.1]	2.6 [2.5;2.6]	***0.001***	3.2 [2.8;3.7]	2.6 [2.6;2.6]	***0.006***
Social	2.6 [2.5;2.7]	2.3 [2.2;2.3]	***< 0.001***	2.6 [2.4;2.7]	2.4 [2.3;2.4]	0.055	2.6 [2.5;2.8]	2.3 [2.3;2.4]	***< 0.001***	2.9 [2.6;3.2]	2.4 [2.3;2.4]	***0.001***
Self-care	2.6 [2.5;2.6]	2.3 [2.2;2.4]	***< 0.001***	2.5 [2.3;2.7]	2.4 [2.3;2.4]	0.394	2.5 [2.4;2.6]	2.4 [2.3;2.4]	***0.018***	3.1 [2.8;3.4]	2.4 [2.3;2.4]	***< 0.001***
Society	3.8 [3.7;3.9]	2.9 [2.8;3.0]	***< 0.001***	3.8 [3.5;4.0]	3.2 [3.1;3.2]	***< 0.001***	3.8 [3.5;4.0]	3.1 [3.1;3.2]	***< 0.001***	4.7 [4.2;5.1]	3.2 [3.1;3.2]	***< 0.001***
Total	19.8 [19.3;20.3]	16.3 [15.9;16.7]	***< 0.001***	19.3 [18.1;20.4]	17.3 [17.0;17.6]	***0.005***	19.4 [18.5;20.4]	17.1 [16.8;17.4]	***< 0.001***	23.9 [22.8;25.0]	17.2 [17.0;17.5]	***< 0.001***

Adjusted means with 95%-confidence intervals resulting from models adjusted for age, gender and centre are presented. A separate model was calculated for any mental disorder, while major depression, anxiety disorders and somatoform disorders were included in one model

The analysis of the WHO-QoL-BREF 2 item-subjective evaluation of QoL showed that most subjects reported a “good” or “very good” QoL (*n* = 1749, 56.6%), 33.4% (*n* = 1032) and 9.9% (*n* = 305) a “bad” or “very bad” QoL. On the 4 QoL domains, the Global WHO-QoL-BREF score was 67.2 ± 18.2.

The distribution of the WHODAS-II Total scores indicated that 24.9% (*n* = 771) reported no disability, 33.9% (*n* = 1048) mild disability, 19.2% (*n* = 594) moderate disability and 22% (*n* = 679) severe disability. The 25th, 50th (median), and 75th percentiles for the mean WHODAS-II scores were 12, 15, and 20, respectively (online supplemental Table 1). Regarding the Self-Rated Health (SRH), most people (62%) reported their overall health to be either “very good” (*n* = 457) or “good” (*n* = 1454). When looking at the individual level of health as self-rated, “Moderate” SRH was reported by 32% and “bad” or “very bad” overall health by 6.3%.

The WHODAS-II was associated with WHO-Qol-BREF Physical scale (β = − 0.14, 95%-CI [− 0.17; − 0.11]; *p* < 0.001), WHO-Qol-BREF Global scale (β = − 0.05, 95%-CI [− 0.07; − 0.03]; *p* < 0.001), and SRH score (β = 1.4, 95%-CI [0.69, 2.17]; *p* = 0.001).

Men reported higher scores on WHO-QoL-BREF global score (unadjusted: 68.54, 95%-CI [67.20; 69.87] vs 66.04, 95%-CI [62.74; 69.34]; p_adjusted_ = 0.015) and lower scores on WHODAS-II (unadjusted: 16.60, 95%-CI [15.68; 17.51] vs 18.24, 95%-CI [17.41; 19.08]; p_adjusted_ < 0.001) than women. On SHR, men indicated a healthier perceived state than women (unadjusted: 2.24, 95%-CI [2.16; 2.32] vs 2.43, 95%-CI [2.31; 2.55]; p_adjusted_ < 0.001) (Table 1).

According to the four age groups (65–69, 70–74, 75–79, 80–84 years), age was related to WHO-QoL-BREF global score, with decreasing levels of QoL with increasing age group (p_linear_ = 0.022). Regarding WHO-QoL-BREF subscales, a significant decreasing effect by increasing age was found for the Physical subscale (p_linear_ < 0.001), no age effect for Social subscale neither linear nor overall group differences (p_linear_ = 0.399, p_group_ = 0.277) and a significant gender and age group interaction (p_interaction_ = 0.011) for Environment subscale, but only group differences no linear age trends in both, males and females (male: p_linear_ = 0.111, p_group_ = 0.012; female: p_linear_ = 0.788, p_group_ = 0.003). Likewise, for the WHODAS-II Global there was a decrease of the LoF by increasing age group (p_linear_ < 0.001), with a significant linear trend on the individual dimensions of the WHODAS-II Mobility (p_linear_ < 0.001), Household (p_linear_ < 0.001), Cognitive (p_linear_ < 0.001), Self-care (p_linear_ < 0.001), and Society (p _linear_ < 0.001), while no age effect was found for the Social domain neither linear nor group (p_linear_ = 0.426, p_group_ = 0.443).

There were significant differences on QoL and LoF between centres (p_group_ < 0.001 each scale).. On the WHO-QoL-BREF Global, Geneva showed the highest levels of QoL while Ferrara, Madrid and Jerusalem did not differ significantly and had the lowest. Looking at the WHO-QoL-BREF subscales a similar order could be observed; for the Physical subscale Geneva and Hamburg had the highest level and the other four did not differ at a lower level; for the Environment subscale Jerusalem showed the lowest level and Geneva again the highest, Ferrara and Madrid did not differ significantly as well as Hamburg and Madrid whereby Ferrara had a significant lower score than Hamburg; for the Social subscale three clusters could be identified Geneva and London with the highest scores, Hamburg and Madrid at the middle and Jerusalem and Ferrara with the lowest scores. For the WHODAS II Global, Geneva showed the highest levels of LoF (lower WHODAS II scores) and Jerusalem the lowest (higher WHODAS II scores) while Hamburg, Ferrara and Madrid shared second place (see Table 1 for details). These data were confirmed in the analysis of all the singles WHODAS II dimensions (mobility, household, cognitive, self-care and society), except the social.

### Mental disorders and QoL / LoF

Adjusted for age, gender and center, elderly who had any ICD-10 mental disorder reported lower levels of QoL and LoF than patients without mental disorders. More specifically the former showed significantly lower scores on the WHO-Qol-BREF (poorer QoL) and higher scores on all the dimensions of the WHODAS-II (lower LoF) than elderly with no mental disorder (all *p* < 0.001). When examining the QoL and LoF scores according to individual psychiatric diagnoses, participants with depression had significantly lower scores on all WHO-Qol-BREF scales and higher on WHODAS-II household (*p* < 0.01), cognitive (*p* = 0.02) and society (*p* < 0.01) subscales than non-depressed; those with anxiety disorders had lower scores on all WHO-QoL-BREF scales (*p* < 0.01) and higher scores on all WHODAS-II scales (between *p* < 0.05 and *p* < 0.01) than non-anxious subjects; and those with somatoform disorders had significantly lower WHO-QoL-BREF scores (*p* < 0.01) and higher scores on all the WHODAS II dimensions (*p* < 0.01) than participants with no somatoform disorder (see Table 2 for details).

### Regression analysis

A series of regression analyses were conducted in order to examine the effects of mental and physical disorders (analysis 1), and subsequently by adding socio-demographic factors (analysis 2), on LoF and QoL.

In analysis 1, scores on WHO-QoL-BREF-Global, indicating poor QoL, were associated with anxiety, depressive or somatoform disorders (all *p* < 0.001), as well as with physical illnesses (i.e. cardiologic, neurological, musculoskeletal, respiratory, genitourinary, endocrinological, cancer, other disorders) (all *p* < 0.001), apart form gastrointestinal and dermatological diseases. Identical associations were shown between the above-said psychiatric and physical disorders, and the WHO-QoL-BREF-Physical Health dimension (all *p* < 0.001). The WHO-QoL-BREF-Social Relationships dimension was associated with all mental disorders (except major depression) (*p* < 0.001) and, among the physical diseases, only neurological disorders (*p* < 0.001). Mental disorders again (except major depression) and some physical disorders (i.e. heart, neurological and musculoskeletal disorders) were significantly associated with the WHO-QoL-BREF-Environment dimension (see Table 3 for details). Regarding disability, poorer LoF (WHODAS-II) was associated with anxiety disorders (b = 0.95 [95% CI 0.02; 1.89], *p* = 0.04), any affective disorders (but not major depression) (b = 2.72 [95% CI 0.75; 4.70], *p* < 0.01), somatoform disorders (b = 4.51[95% CI 3.22; 5.81], *p* < 0.001), cardiologic (b = 1.09 [95%CI 0.60; 1.57] *p* < 0.001), musculoskeletal (b = 2.27 [95% CI 1.50; 3.04], *p* < 0.001), neurological (b = 2.57 [95%CI 1.39; 3.74], *p* < 0.001), respiratory (b = 1.68 [95%CI 0.75; 2.61], *p* = 0.001), genitourinary (b = 1.63 [95%CI 0.41; 2.86], *p* = 0.012) and other physical diseases (b = 2.16 [95%CI 1.17; 3.15], *p* < 0.001) (Table 3).

**Table 3:** Regression analysis of mental and physical disorders on WHO-QoL Bref and WHODAS II

	WHODAS II		WHOQoL-BREF							
	TOTAL		PHYSICAL HEALTH		SOCIAL RELATIONSHIP		ENVIRONMENT		GLOBAL SCORE	
	b [95%-CI]	*p*-value	b [95%-CI]	*p*-value	b [95%-CI]	*p*-value	b [95%-CI]	*p*-value	b [95%-CI]	*p*-value
**Any Anxiety Disorder (Year)** (ref. No)										
- Yes	0.95 [0.02; 1.89]	***0.046***	−6.40 [− 8.69; −4.12]	***< 0.001***	− 3.75 [− 5.14; − 2.36]	***< 0.001***	−1.96 [− 3.66; − 0.26]	***0.026***	− 3.76 [− 6.34; − 1.18]	***0.006***
**Any Affective Disorder (Year)** (ref. No)										
- Yes	2.72 [0.75; 4.70]	***0.009***	− 10.64 [− 15.01; − 6.27]	***< 0.001***	− 10.78 [− 16.76; − 4.81]	***0.001***	− 5.74 [− 10.58; − 0.91]	***0.022***	−13.79 [− 18.79; − 8.79]	***< 0.001***
**Any Major Depressive Disorder (Year)** (ref. No)										
- Yes	− 1.71 [− 3.59; 0.17]	0.073	7.04 [2.71; 11.37]	***0.003***	4.33 [− 4.29; 12.96]	0.307	4.74 [− 0.91; 10.39]	0.095	11.22 [4.74; 17.71]	***0.002***
**Any Somatoform Disorder (Year)** (ref. No)										
- Yes	4.51 [3.22; 5.81]	< ***0.001***	−8.81 [−12.15; − 5.46]	***< 0.001***	− 4.43 [− 7.82; − 1.04]	0.***013***	− 6.41 [− 11.70; − 1.13]	***0.020***	−8.71 [− 11.94; − 5.48]	***< 0.001***
**Any Mental Disorder - Without Nicotine Dependence (Year)** (ref. No)										
- Yes	0.92 [− 0.07; 1.91]	0.067	0.82 [−1.52; 3.17]	0.472	0.26 [−1.38; 1.89]	0.747	−1.38 [− 3.94; 1.17]	0.272	− 0.54 [− 2.81; 1.74]	0.629
**Any Heart Disorder** (ref. No)										
- Yes	1.09 [0.60; 1.57]	***< 0.001***	− 5.52 [− 7.02; − 4.02]	***< 0.001***	− 0.58 [− 2.16; 1.00]	0.454	−1.73 [− 2.91; − 0.55]	***0.006***	− 3.17 [− 5.65; − 0.68]	***0.015***
**Any CNS Disorder** (ref. No)										
- Yes	2.57 [1.39; 3.74]	***< 0.001***	− 7.58 [− 9.87; − 5.28]	***< 0.001***	− 3.55 [− 6.25; − 0.84]	0.013	− 3.66 [− 5.35; − 1.96]	***< 0.001***	−7.83 [− 9.65; − 6.01]	***< 0.001***
**Any Musculoskeletal Disorder** (ref. No)										
- Yes	2.27 [1.50; 3.04]	***< 0.001***	−6.76 [− 8.70; − 4.83]	***< 0.001***	−1.50 [− 3.02; 0.02]	0.053	− 2.27 [− 3.83; − 0.70]	***0.007***	−4.73 [− 5.97; − 3.49]	***< 0.001***
**Any Respiratory Disorder** (ref. No)										
- Yes	1.68 [0.75; 2.61]	***0.001***	−5.63 [− 7.70; − 3.57]	***< 0.001***	−2.38 [− 4.80; 0.05]	0.054	−1.22 [− 3.50; 1.07]	0.280	−6.17 [− 8.84; − 3.50]	***< 0.001***
**Any Gastrointestinal Disorder** (ref. No)										
- Yes	−0.28 [− 1.30; 0.74]	0.577	0.29 [− 3.48; 4.07]	0.873	0.83 [− 1.39; 3.06]	0.443	− 0.11 [− 1.90; 1.67]	0.895	− 2.05 [− 4.28; 0.17]	0.068
**Any Genitourinary Disorder** (ref. No)										
- Yes	1.63 [0.41; 2.86]	***0.012***	−4.28 [− 7.64; − 0.91]	***0.015***	−2.34 [− 5.53; 0.86]	0.143	− 1.12 [− 3.40; 1.16]	0.317	− 3.45 [− 6.02; − 0.87]	***0.011***
**Any Endocrinological Disorder** (ref. No)										
- Yes	0.38 [− 0.25; 1.02]	0.222	−5.42 [− 8.24; − 2.61]	***0.001***	−0.61 [− 2.36; 1.14]	0.477	− 0.73 [− 2.17; 0.70]	0.299	− 3.62 [− 5.20; − 2.04]	***< 0.001***
**Any Cancer Disorder** (ref. No)										
- Yes	0.02 [− 1.23; 1.27]	0.977	−5.21 [−9.42; − 0.99]	***0.018***	0.02 [−2.61; 2.66]	0.987	0.79 [−2.94; 4.52]	0.663	−3.99 [−7.29; − 0.69]	***0.020***
**Any Dermatological Disorder** (ref. No)										
- Yes	1.28 [− 0.14; 2.71]	0.075	−2.15 [− 6.27; 1.98]	0.290	−0.66 [− 4.68; 3.36]	0.735	− 0.53 [− 3.07; 2.01]	0.670	0.29 [− 3.26; 3.84]	0.866
**Any Other Disorder** (ref. No)										
- Yes	2.16 [1.17; 3.15]	***< 0.001***	− 6.47 [− 10.00; − 2.93]	***0.001***	−0.05 [− 2.36; 2.25]	0.962	0.30 [−1.55; 2.15]	0.736	−4.02 [− 7.60; − 0.44]	0.***030***

In the second set of analysis socio-demographic variables were added. The WHO-Qol-BREF Physical health dimension, a worsening financial situation (“poor” to “good” in comparison with “very good”) (p = 0.01), frequency of financial problems (“always” and “often” in comparison to “never”) (*p* < 0.01), having anxiety, depressive and somatoform disorders (*p* ≤ 0.01) and a number of physical disorders (i.e. heart, neurological, respiratory, endocrinological, musculoskeletal, *p* < 0.01; genitourinary and cancer, *p* < 0.05), were negative predictors, whereas being working situation (“employed” in comparison to “retired”), was positive predictor of better QoL (*p* < 0.01) (see online supplemental Table 2). Financial situation (“just enough” and “poor” versus “very good”) (*p* < 0.01), frequency of financial problems (“rarely” in comparison to “never”) (*p* < 0.01), number of children (*p* < 0.01), major depression (*p* < 0.05), neurological and cancer (*p* < 0.05) predicted lower WHO-QoL-BREF-Social relationship scale; an increasing number of current close significant predicted higher level of QoL (*p* < 0.01). WHO-QoL-BREF-Environment was negatively predicted by financial situation (“just enough” and “poor” versus “very good”) (*p* < 0.05), a diagnosis of somatoform disorders (*p* < 0.01), and physical diagnoses (i.e. heart, *p* < 0.01; neurological, musculoskeletal disorders, *p* < 0.05), while rate of burden of care (“no burden” *p* < 0.01, or “some burden” *p* < 0.05, in comparison to “a lot of burden”) resulted in a higher QoL. WHO-QoL-BREF-Global was predicted by work status (“homemaker/housewife” in comparison to “retired”) (*p* < 0.05), financial situation (“poor”, *p* < 0.001, “just enough”, *p* < 0.01, “good”, *p* < 0.05, in comparison with “very good”), psychiatric disorders (anxiety, and somatoform disorders, *p* < 0.01; depressive disorders, *p* < 0.05), and a number of physical disorders (i.e. heart, neurological, musculoskeletal, respiratory, endocrinological, and cancer) (*p* < 0.01), resulted in poorer WHO-QoL-BREF-Global, while marital status (“married” or “separated/divorced/widowed” in comparison to “never been married”) (*p* < 0.05), rate of burden of care (“no degree of burden” in comparison to “a lot of burden”) (*p* < 0.01) resulted in better QoL (see online supplemental Table 2).

Regarding the WHODAS-II, work status of participant (“homemaker/housewife” in comparison to “retired”) (*p* = 0.018),worsening financial situation (“poor”-“good” in comparison with “very good”) (*p* < 0.01), frequency of financial problems (“always” in comparison to “never”), plus having any mental disorder (*p* = 0.04) (in particular somatoform disorders, *p* < 0.001), and physical disorders (i.e. heart, neurological, respiratory, genitourinary, endocrinological, musculoskeletal) (*p* ≤ 0.01) were associated with low LoF, while work status of the partner (“employed” in comparison to “retired”) (*p* = 0.04), religious affiliation (“not very important” in comparison to “very important”) (*p* = 0.01), and number of current close significant others (*p* = 0.03) were predictors of better LoF (see online supplemental Table 2 for details).

## Discussion

Against the background of an ageing European society and the need to gain more knowledge about the mental health, disability and quality of life of the elderly population, this study presents data on QoL and LoF and its relationship with mental disorders and other variables in six European countries, as part of the MentDis65+ project.

The first finding of our study is that not a high percentage of elderly people participating in the survey reported poor levels of QoL or LoF. This result indicate that general condition of QoL and LoF in the elderly seem sufficiently preserved in Europe, although less preserved with respect to younger population [5, 6, 29]. The mean score of the WHO-QoL-BREF of the elderly population (67.2 ± 18.2) was higher than the score that has been considered adequately detecting poor QoL (score < 60). With regard to disability, the distribution of the WHODAS-II scores resemble the data of both a Canadian community sample [30] and of a Portuguese study within the World Mental Health Survey Initiative Portugal [31], although, again, our sample of elderly subjects had lower levels of LoF.

Some dimensions of QoL and LoF tended to decrease with age, when analysis was done in different age groups (65–69; 70–74; 75–79; 80–84 years). Regarding QoL, age was associated with lower levels of global physical and environmental QoL. Likewise, lower levels of functioning in the oldest population were found to be in the dimensions of understanding and communicating, general life activities, getting around, self-car, and global LoF. These results, calling for attention to the oldest members of the population, are in line with the English Longitudinal Study of Ageing on a population aged 50 and over, in which gender, education, depression, limiting long-standing illness, difficulty with ADL-s, lack of wealth, non-employment, decreased number of friends and low positive support had a negative impact on QoL [5, 6]. These figures also confirm what emerged in the only other available multicenter European study (ESEMeD) [16–18] investigating QoL and LoF among adult population, of which only a small part however regarded elderly people.

Female gender and poor education were associated with low levels of QoL and LoF, indicating that socio-demographic and lifestyle factors seem to be influential factors [32]. This finding would merit however to be better explored in future studies, since, in spite of their reported QoL and LoF, the expectation of life of men is reported to be lower than women, although the mortality of women is also increased by a series of health factors reducing QoL [33] Education has also been found to be significantly associated with better QoL when comparing developing countries to developed countries, in a study of non-elderly population [34]. This should be considered as an important aspect for future research and clinical practice, given immigration movements in Europe over the last 25 years and the possible effects on psychiatric morbidity and QoL, since inequalities in housing, social support, income and physical health status have been shown between elderly immigrants and local residents [35].

There were some differences in both QoL and LoF across study centres. Switzerland reported higher levels of QoL (physical and environment) and LoF (WHODAS II Global) as compared to other countries, especially Israel, that had the lowest. Higher levels of QoL (social) were found again in Switzerland, and also in England. Interpretation of these results is not easy, however. The explanation of these differences is not easy. As underlined by some authors, [36] it is always difficult, in multicenter studies, to interpret possible differences among countries, since different response styles, differences in the meaning of response levels within the various language versions of the questionnaires or, cultural variables, play a possible role in molding the impact on the people’s perception of their own QoL and LoF. In agreement with this, data from the European ESEMeD study have shown that among 1659 respondents aged ≥75 years recruited from Belgium, France, Germany, Italy, Netherlands, and Spain, there were significant differences between countries with the lowest QoL scores in Italy and the highest in the Netherlands, although it was not possible to completely understand the reasons for these differences, due to the many variables involved including the different health organizational systems in the countries [17].. On the other hand, significant differences in QoL and LoF, as ascertained by using the WHO-QoL-BREF and the WHODAS II, were also found between Asian and African countries, [37] as well as in low-income countries, with differences not fully explained by age, socioeconomic status, marital status, education or other health factors [38]. However, the data call for attention at the European Union about the need for governmental policies in order to provide more homogeneous health care to the population that is aging amongst the different countries members.

A second finding of the study was that having an ICD-10 psychiatric diagnosis was associated with substantial levels of disability and loss of QoL, across all the dimensions measured by the WHO-QoL-BREF and the WHODAS-II. Elderly people who were cases of depression, anxiety and somatoform disorders had lower levels of QoL in general and in the single dimensions regarding the presence of symptoms of pain and discomfort, energy and fatigue (physical), problems with one’s own environment, recreation and leisure (environment) and interpersonal relationships (social). Regarding specifically the WHODAS-II, disability was related to psychiatric morbidity, especially affective disorders, anxiety and somatoform disorders.

All these data add a significant aspect in elderly people, since it is a confirmation of studies carried out in adult non-elderly population in Europe Australia [39], Sweden [40], Canada [29], Portugal [30] and Europe [41] indicating that having common mental disorders (i.e. those are not usually classified as major mental disorders, such as psychosis or other severe mental disorders) has also an impact on disability and QoL. This can be interpreted as a possible tendency of people with psychiatric problems, especially depression, to have a negatively biased view on the world, therefore of their QoL, as recently underlined as a caveat when measuring community-dwelling older adults’ QOL and providing active ageing programs [42]. However, literature has also underlined that depression, self-care ability, and medical care burden are independent factors molding the quality of life of the elderly [14, 43, 44], as also confirmed in another Ment_Dis65+ study specifically dealing with depressive disorders in the elderly [45].

As expected and in agreement with studies showing a concomitant and reciprocal role of physical and mental disorders in its association with loss of QoL [46], suffering from medical disorders diagnosable in the sample, such as cancer, heart and respiratory diseases, endocrinological disorders, was also a factor related to both disability and reduction of QoL. The role of mental disorders in negatively influencing the QoL and the LoF of the subjects was maintained, although in a smaller way, when other variables were considered. In fact, when in regression analysis socio-demographic variables were added, some other factors (namely retirement, worsening of financial situation, frequency of financial problems) in entered the equation, together with physical disorders in reducing both QoL and LoF. Taken together, the data support the view that the maintenance of good QoL and LoF in older aged people is enabled by reducing the incidence of mental disorders, helping people to maintain physical function and avoid sedentary behavior [47], and having social relationship [29, 48]. These issues should be considered in the light of a previous report of the MentDis_ICF65+ study which has shown that 47% of the subjects had experienced a mental disorder in their lifetime, about 30% within the past year and about 25% had a current mental disorder, especially anxiety disorders and affective disorders [49].

A number of limitations should be mentioned in interpreting the results of this study. The first concerns the representativeness of the sample. The exclusion of people with cognitive impairment limits the generalizability of our findings, since it has been shown that older adults with mild cognitive impairment report poorer QoL than those who are not cognitively impaired [50]. Second, more detailed investigation and analysis of other factors, such as concomitant medications, performance levels, and social and family support, as well as spiritual and religious affiliation that can influence QoL and LoF among the elderly [51], would have been necessary. Third, the statistical and the relative results should be interpreted strictly explorative, although other analyses (e.g. structural equation modeling needing the definition of a non data-driven theoretical model of the variables’ dependence structure) were considered, but not maybe proper in our case. Also, the cross-sectional nature of this study does not allow a more comprehensive understanding of the changes of the outcomes, namely QoL and LoF, across time, as it will be done when prospectively analyzing the MentDis_ICF65 data. Last, since impairment in QoL and LoF increases with age, more information should be available on subjects over 85, that the Ment_Dis65+ did not take into consideration in its design [19], given the data regarding the poorer QoL in old-old individuals [5, 52, 53].

## Conclusions

The main findings of this study were that QoL and LoF were quite acceptable in older Europeans, but also that a series of variables were associated with low levels of both QoL and LoF, including both physical and mental disorders which are common in the elderly (i.e. depression, anxiety, somatoform disorders) as well socio-demographic factors. There were also some difference among centers (namely Switzerland vs Israel) that could be related to possible cultural biases, as emerged in other studies of non-elderly population, or to different subjective perception of health in the participating countries.

In general, what emerges is the need for attention to the vulnerable segment of the population, such as the elderly, and the importance of taking into account the variables that influence in a negative way the QoL and the LoF. Setting up specific mental health services for elderly people and the implementation of collaborative liaison between mental health, social and community and primary care health services [54] in order to provide specific and person-centered intervention for those showing low levels of QoL or disability is mandatory in European countries.

Despite these limitations, we assume that elderly individuals with depression are more impaired than elderly individuals without a depressive disorder. Furthermore, our results show that adequate interventions for the majority of older depressed individuals are lacking.

One major strength of this study stems from the development and use of a reliable and valid age-specific structured-standardized interview, which has resulted in higher prevalence rates that illustrate the need for policy for one of the most frequent mental disorders in old age.

Additional studies could also integrate primary care perspectives into the diagnostics as this is where the majority of older adults with mental health problems are treated; thus, this will enable the general practitioner to give advice on specialised mental healthcare. Finally, further studies should examine whether the use of services corresponds to the high burden of mental illness in elderly people.

## Supplementary information

**Additional file 1: Online supplementary Table 1.** Percentile distribution of the WHODS-II scores

**Additional file 2: Online supplemental Table 2.** Regression analysis of the socio-demographic, mental and physical disorders on WHODAS II and WHO-QoL Bref (adjusted for age group, sex (and interaction if significant) as well as study centre)

## Data Availability

Not applicable.

## Abbreviations

CAPI: Computer-assisted personal interviewing
ESEMeD: European study of the epidemiology of mental disorders
ICD-10: International classification of disease 10th edition
LoF: Level of functioning
MentDis_ICF65 +: Prevalence, 1-year incidence and symptom severity of mental disorders in the elderly Relationship to impairment, functioning (ICF) and service utilization".
QoL: Quality of life
SHR: Self-health rated
WHODAS-II: World health organization disability assessment schedule -II
WHO-QoL-BREF: World health organization quality of life brief
