# Supplementary material for: Quality of life, level of functioning, and its relationship with mental and physical disorders in the elderly: results from the MentDis_ICF65+ study
Source: Health Qual Life Outcomes. 2020 Mar 6;18:61. doi: 10.1186/s12955-020-01310-6 (PMC7060594; doi:10.1186/s12955-020-01310-6)
Supplement: Supplementary file 1 — Additional file 1: Online supplementary Table 1. Percentile distribution of the WHODS-II scores [file 12955_2020_1310_MOESM1_ESM.docx]

**Online supplementary Table 1.** Percentile distribution of the WHODS-II scores

|  |  | **Overall** | **Gender** | | **Age** | | | | **Study centre** | | | | | |
| --- | --- | --- | --- | --- | --- | --- | --- | --- | --- | --- | --- | --- | --- | --- |
|  |  |  | **Male** | **Female** | **65-69** | **70-74** | **75-79** | **80-84** | **Hamburg** | **London** | **Madrid** | **Ferrara** | **Geneva** | **Jerusalem** |
| **WHODAS II** | |  |  |  |  |  |  |  |  |  |  |  |  |  |
| Total Score | 25th | 12 | 12 | 13 | 12 | 12 | 13 | 14 | 13 | 13 | 12 | 12 | 12 | 14 |
|  | 50th | 15 | 14 | 16 | 14 | 15 | 16 | 19 | 15 | 16 | 15 | 14 | 14 | 18 |
|  | 75th | 20 | 18 | 22 | 17 | 20 | 21 | 25 | 20 | 23 | 19 | 19 | 18 | 26 |
|  | 90th | 28 | 25 | 29 | 24 | 27 | 27 | 33 | 27 | 30 | 26 | 28 | 22 | 35 |
|  | 95th | 32 | 32 | 32 | 29 | 30 | 30 | 37 | 31 | 34 | 30 | 32 | 26 | 39 |
| Mobility | 25th | 2 | 2 | 2 | 2 | 2 | 2 | 2 | 2 | 2 | 2 | 2 | 2 | 2 |
|  | 50th | 3 | 2 | 3 | 2 | 3 | 3 | 5 | 3 | 4 | 2 | 3 | 2 | 4 |
|  | 75th | 5 | 4 | 6 | 4 | 5 | 6 | 8 | 5 | 6 | 5 | 5 | 4 | 6 |
|  | 90th | 8 | 7 | 8 | 6 | 7 | 8 | 9 | 7 | 8 | 7 | 8 | 6 | 9 |
|  | 95th | 9 | 8 | 9 | 8 | 8 | 9 | 10 | 9 | 9 | 8 | 9 | 8 | 10 |
| Household | 25th | 2 | 2 | 2 | 2 | 2 | 2 | 2 | 2 | 2 | 2 | 2 | 2 | 2 |
|  | 50th | 2 | 2 | 2 | 2 | 2 | 2 | 3 | 2 | 2 | 2 | 2 | 2 | 3 |
|  | 75th | 4 | 3 | 4 | 3 | 3 | 4 | 5 | 4 | 4 | 4 | 3 | 3 | 5 |
|  | 90th | 6 | 5 | 6 | 5 | 5 | 5 | 7 | 6 | 6 | 5 | 5 | 5 | 7 |
|  | 95th | 7 | 6 | 7 | 6 | 6 | 6 | 8 | 7 | 6 | 6 | 8 | 6 | 8 |
| Cognitive | 25th | 2 | 2 | 2 | 2 | 2 | 2 | 2 | 2 | 2 | 2 | 2 | 2 | 2 |
|  | 50th | 2 | 2 | 2 | 2 | 2 | 2 | 2 | 2 | 2 | 2 | 2 | 2 | 2 |
|  | 75th | 3 | 3 | 3 | 2 | 3 | 3 | 4 | 3 | 3 | 3 | 2 | 3 | 4 |
|  | 90th | 4 | 4 | 4 | 4 | 4 | 4 | 5 | 4 | 5 | 4 | 4 | 4 | 6 |
|  | 95th | 5 | 5 | 5 | 5 | 5 | 5 | 6 | 5 | 6 | 5 | 5 | 4 | 7 |
| Social | 25th | 2 | 2 | 2 | 2 | 2 | 2 | 2 | 2 | 2 | 2 | 2 | 2 | 2 |
|  | 50th | 2 | 2 | 2 | 2 | 2 | 2 | 2 | 2 | 2 | 2 | 2 | 2 | 2 |
|  | 75th | 2 | 2 | 2 | 2 | 2 | 2 | 2 | 2 | 2 | 2 | 2 | 2 | 3 |
|  | 90th | 4 | 4 | 3 | 3 | 4 | 4 | 3 | 4 | 4 | 3 | 3 | 3 | 5 |
|  | 95th | 4 | 5 | 4 | 4 | 5 | 4 | 4 | 4 | 4 | 4 | 4 | 4 | 6 |
| Self-care | 25th | 2 | 2 | 2 | 2 | 2 | 2 | 2 | 2 | 2 | 2 | 2 | 2 | 2 |
|  | 50th | 2 | 2 | 2 | 2 | 2 | 2 | 2 | 2 | 2 | 2 | 2 | 2 | 2 |
|  | 75th | 2 | 2 | 2 | 2 | 2 | 2 | 3 | 2 | 2 | 2 | 2 | 2 | 2 |
|  | 90th | 3 | 3 | 3 | 2 | 3 | 3 | 6 | 3 | 4 | 3 | 3 | 2 | 5 |
|  | 95th | 5 | 5 | 5 | 4 | 4 | 5 | 7 | 4 | 6 | 4 | 6 | 3 | 8 |
| Society | 25th | 2 | 2 | 2 | 2 | 2 | 2 | 2 | 2 | 2 | 2 | 2 | 2 | 2 |
|  | 50th | 3 | 2 | 3 | 2 | 3 | 3 | 3 | 3 | 3 | 2 | 2 | 2 | 3 |
|  | 75th | 4 | 4 | 4 | 4 | 4 | 4 | 5 | 4 | 4 | 4 | 4 | 4 | 5 |
|  | 90th | 6 | 5 | 6 | 5 | 5 | 5 | 6 | 6 | 5 | 5 | 6 | 5 | 7 |
|  | 95th | 6 | 6 | 7 | 6 | 6 | 6 | 7 | 6 | 6 | 6 | 7 | 6 | 8 |
