# Supplementary material for: Quality of life, level of functioning, and its relationship with mental and physical disorders in the elderly: results from the MentDis_ICF65+ study
Source: Health Qual Life Outcomes. 2020 Mar 6;18:61. doi: 10.1186/s12955-020-01310-6 (PMC7060594; doi:10.1186/s12955-020-01310-6)
Supplement: Supplementary file 2 — Additional file 2: Online supplemental Table 2. Regression analysis of the socio-demographic, mental and physical disorders on WHODAS II and WHO-QoL Bref (adjusted for age group, sex (and interaction if significant) as well as study centre) [file 12955_2020_1310_MOESM2_ESM.docx]

Online supplemental Table 2. Regression analysis of the socio-demographic, mental and physical disorders on WHODAS II and WHO-QoL Bref (adjusted for age group, sex (and interaction if significant) as well as study centre)

|  | WHODAS II | | WHOQoL-BREF | | | | | | | |
| --- | --- | --- | --- | --- | --- | --- | --- | --- | --- | --- |
|  | TOTAL | | PHYSICAL HEALTH | | SOCIAL RELATIONSHIP | | ENVIRONMENT | | GLOBAL SCORE | |
|  | b  [95%-CI] | p-value | b  [95%-CI] | p-value | b  [95%-CI] | p-value | b  [95%-CI] | p-value | b  [95%-CI] | p-value |
| **Parents Born In Same Country** (ref. No) |  |  |  |  |  |  |  |  |  |  |
| - Yes | 1.18  [-0.04; 2.41] | ***0.057*** | -0.49  [-7.94; 6.96] | 0.892 | -3.63  [-8.03; 0.76] | 0.100 | -0.87  [-4.45; 2.70] | 0.616 | -2.72  [-6.17; 0.74] | 0.117 |
| **Marital Status (3 Categories)** (ref. Never Been Married/Other) |  |  |  |  |  |  |  |  |  |  |
| - Married | -6.73  [-20.17; 6.72] | 0.309 | 8.69  [-1.57; 18.96] | 0.093 | -3.33  [-21.72; 15.07] | 0.710 | 4.14  [-2.83; 11.10] | 0.230 | 7.48  [1.48; 13.49] | ***0.017*** |
| - Separated/Divorced/Widowed | -6.20  [-19.71; 7.32] | 0.350 | 6.69  [-4.30; 17.68] | 0.219 | 0.21  [-17.96; 18.38] | 0.981 | 3.15  [-4.52; 10.83] | 0.402 | 8.15  [1.00; 15.31] | ***0.028*** |
| **Work Status** (ref. Retired) |  |  |  |  |  |  |  |  |  |  |
| - Homemaker/Housewife | 1.06  [0.20; 1.92] | ***0.018*** | -0.93  [-3.52; 1.65] | 0.460 | 1.74  [-2.41; 5.89] | 0.392 | 1.38  [-1.27; 4.03] | 0.290 | -4.40  [-8.41; -0.40] | ***0.033*** |
| - Working/Employed | -0.34  [-1.40; 0.72] | 0.512 | 3.95  [1.06; 6.84] | ***0.010*** | 1.98  [-0.81; 4.77] | 0.154 | -0.69  [-3.84; 2.47] | 0.654 | -2.13  [-5.91; 1.64] | 0.252 |
| - Other | -1.00  [-2.79; 0.79] | 0.259 | 1.86  [-4.65; 8.37] | 0.558 | 0.71  [-5.04; 6.46] | 0.800 | -3.94  [-14.77; 6.89] | 0.457 | 2.09  [-5.78; 9.96] | 0.586 |
| **Work Status Of Spouse/Partner** (ref. Retired) |  |  |  |  |  |  |  |  |  |  |
| - Homemaker/Housewife | 0.21  [-0.62; 1.04] | 0.605 | -1.94  [-5.37; 1.49] | 0.251 | 0.55  [-0.95; 2.04] | 0.455 | -0.19  [-3.11; 2.73] | 0.892 | -1.03  [-2.73; 0.68] | 0.223 |
| - Working/Employed | -0.91  [-1.78; -0.04] | ***0.042*** | 1.98  [-0.80; 4.76] | 0.153 | 0.10  [-2.92; 3.11] | 0.947 | -0.01  [-3.76; 3.74] | 0.997 | 1.93  [-1.52; 5.37] | 0.257 |
| - Unemployed | -0.89  [-4.07; 2.30] | 0.568 | -0.09  [-7.06; 6.87] | 0.978 | -12.51  [-25.75; 0.73] | 0.063 | -0.69  [-6.82; 5.45] | 0.818 | -6.15  [-18.32; 6.01] | 0.304 |
| - Other | -0.51  [-2.86; 1.83] | 0.652 | 3.91  [-3.80; 11.63] | 0.302 | -2.51  [-13.76; 8.75] | 0.647 | 1.11  [-7.42; 9.63] | 0.790 | 2.37  [-6.41; 11.15] | 0.579 |
| **Rate Degree Of Care Burden** (ref. A Lot Of Burden) |  |  |  |  |  |  |  |  |  |  |
| - No Burden | -1.01  [-2.81; 0.79] | 0.256 | 0.20  [-7.06; 7.46] | 0.955 | 3.01  [-1.05; 7.07] | 0.138 | 5.98  [2.21; 9.75] | ***0.003*** | 5.68  [1.65; 9.70] | ***0.008*** |
| - Some Burden | -0.80  [-2.79; 1.20] | 0.417 | 1.23  [-7.82; 10.28] | 0.780 | 0.91  [-3.87; 5.69] | 0.696 | 6.53  [1.20; 11.87] | ***0.019*** | 4.12  [-1.56; 9.80] | 0.146 |
| **Financial Situation** (ref. Very Good) |  |  |  |  |  |  |  |  |  |  |
| - Good | 1.49  [0.52; 2.46] | ***0.004*** | -6.68  [-8.85; -4.52] | ***<0.001*** | -3.29  [-7.46; 0.89] | 0.116 | -4.25  [-8.63; 0.12] | 0.056 | -4.57  [-8.43; -0.71] | ***0.023*** |
| - Just Enough | 2.76  [1.69; 3.84] | ***<0.001*** | -9.34  [-13.85; -4.83] | ***<0.001*** | -6.03  [-9.95; -2.12] | ***0.004*** | -6.28  [-11.77; -0.79] | ***0.027*** | -8.04  [-12.99; -3.08] | ***0.003*** |
| - Poor | 5.33  [2.93; 7.72] | ***<0.001*** | -14.10  [-19.77; -8.43] | ***<0.001*** | -7.59  [-11.85; -3.33] | ***0.001*** | -9.14  [-15.93; -2.35] | ***0.011*** | -14.70  [-20.76; -8.63] | ***<0.001*** |
| - Very Poor | -1.58  [-5.70; 2.55] | 0.435 | -6.38  [-25.66; 12.91] | 0.498 | -14.23  [-30.10; 1.65] | 0.076 | -4.04  [-14.51; 6.43] | 0.430 | -13.39  [-28.51; 1.73] | 0.080 |
| **Frequency Of Financial Problems** (ref. Never) |  |  |  |  |  |  |  |  |  |  |
| - Rarely | -0.18  [-1.05; 0.68] | 0.663 | -1.89  [-4.25; 0.47] | 0.110 | -1.87  [-3.04; -0.71] | ***0.003*** | -0.89  [-2.66; 0.87] | 0.303 | -1.57  [-3.65; 0.51] | 0.131 |
| - Often | 0.36  [-1.93; 2.65] | 0.747 | -5.65  [-8.77; -2.53] | ***0.001*** | -1.28  [-5.37; 2.81] | 0.521 | -0.54  [-5.12; 4.03] | 0.806 | -4.91  [-11.81; 1.98] | 0.153 |
| - Always | 4.30  [1.24; 7.36] | ***0.008*** | -13.46  [-21.39; -5.53] | ***0.002*** | -3.50  [-10.17; 3.17] | 0.287 | -4.28  [-11.11; 2.55] | 0.206 | -7.62  [-17.28; 2.05] | 0.116 |
| **Religious Affiliation** (ref. Very Important) |  |  |  |  |  |  |  |  |  |  |
| - Somewhat Important | -0.40  [-1.45; 0.64] | 0.430 | 0.75  [-2.23; 3.72] | 0.606 | -0.12  [-2.22; 1.97] | 0.905 | -2.80  [-5.40; -0.21] | 0.035 | -0.89  [-3.55; 1.77] | 0.492 |
| - Not Very Important | -1.13  [-2.02; -0.24] | ***0.015*** | 1.06  [-2.74; 4.86] | 0.566 | -2.13  [-4.68; 0.43] | 0.098 | -2.55  [-5.17; 0.07] | 0.056 | -1.22  [-3.73; 1.29] | 0.324 |
| - Not At All | -0.75  [-1.87; 0.37] | 0.179 | 0.40  [-1.50; 2.31] | 0.663 | -1.41  [-4.64; 1.82] | 0.374 | -2.51  [-5.85; 0.83] | 0.133 | -0.32  [-3.77; 3.12] | 0.847 |
| **Years Of Schooling (Cut Off 13 Years)** | -0.11  [-0.30; 0.09] | 0.266 | 0.37  [-0.04; 0.79] | 0.074 | 0.17  [-0.22; 0.57] | 0.370 | 0.15  [-0.26; 0.56] | 0.459 | 0.28  [-0.13; 0.70] | 0.166 |
| **Number Of Household Members (Number Of People) Sum Score** | 0.36  [-0.15; 0.86] | 0.153 | -0.62  [-2.95; 1.72] | 0.587 | -0.60  [-3.33; 2.13] | 0.653 | -0.18  [-3.20; 2.83] | 0.900 | 0.55  [-0.76; 1.85] | 0.393 |

| **Number Of Children** | 0.05  [-0.44; 0.54] | 0.829 | -0.43  [-1.37; 0.51] | 0.349 | -1.02  [-1.57; -0.46] | ***0.001*** | -0.25  [-1.34; 0.84] | 0.638 | -0.55  [-1.23; 0.13] | 0.107 |
| --- | --- | --- | --- | --- | --- | --- | --- | --- | --- | --- |
| **Number Of Grandchildren** | 0.05  [-0.10; 0.21] | 0.485 | 0.23  [-0.27; 0.73] | 0.343 | 0.23  [-0.15; 0.61] | 0.230 | -0.03  [-0.46; 0.41] | 0.898 | 0.33  [-0.09; 0.74] | 0.118 |
| **Number Of Current Close Significant (Cut Off 50 People)** | -0.04  [-0.08; -0.00] | ***0.032*** | 0.07  [-0.08; 0.22] | 0.340 | 0.22  [0.12; 0.31] | <***0.001*** | 0.03  [-0.07; 0.13] | 0.573 | 0.09  [-0.03; 0.21] | 0.151 |
| **Number Of Different Sources Of Household Income Sum Score** | -0.24  [-0.65; 0.17] | 0.231 | -0.96  [-2.70; 0.78] | 0.263 | -0.73  [-2.39; 0.92] | 0.364 | 0.19  [-1.25; 1.62] | 0.787 | 0.08  [-1.10; 1.26] | 0.888 |
| **Any Anxiety Disorder (Year)** (ref. No) |  |  |  |  |  |  |  |  |  |  |
| - Yes | 0.63 [-1.05; 2.31] | 0.444 | -5.92 [-10.05; -1.79] | ***0.007*** | 1.73 [-0.72; 4.18] | 0.156 | -2.16 [-5.99; 1.67] | 0.253 | -6.26 [-10.32; -2.20] | ***0.004*** |
| **Any Affective Disorder (Year)** (ref. No) |  |  |  |  |  |  |  |  |  |  |
| - Yes | 0.81 [-2.04; 3.67] | 0.559 | -8.06 [-13.63; -2.50] | ***0.007*** | -3.19 [-8.82; 2.45] | 0.252 | -0.93 [-7.58; 5.71] | 0.773 | -12.81 [-23.59; -2.03] | ***0.022*** |
| **Any Major Depressive Disorder (Year)** (ref. No) |  |  |  |  |  |  |  |  |  |  |
| - Yes | -0.27 [-2.69; 2.15] | 0.818 | 3.60 [-2.16; 9.37] | 0.207 | -3.55 [-6.90; -0.20] | ***0.039*** | -2.94 [-7.50; 1.62] | 0.194 | 8.28 [-2.47; 19.04] | 0.124 |
| **Any Somatoform Disorder (Year)** (ref. No) |  |  |  |  |  |  |  |  |  |  |
| - Yes | 5.34 [3.40; 7.29] | ***0.001*** | -8.10 [-14.57; -1.63] | ***0.017*** | -4.04 [-8.46; 0.38] | 0.071 | -7.72 [-12.75; -2.69] | ***0.004*** | -7.58 [-10.99; -4.17] | ***<0.001*** |
| **Any Mental Disorder - Without Nicotine Dependence (Year)** (ref. No) |  |  |  |  |  |  |  |  |  |  |
| - Yes | 1.36 [0.02; 2.69] | ***0.046*** | 0.28 [-2.10; 2.67] | 0.806 | -1.63 [-4.15; 0.89] | 0.193 | -0.61 [-2.87; 1.65] | 0.579 | 1.06 [-2.27; 4.38] | 0.516 |
| **Any Heart Disorder** (ref. No) |  |  |  |  |  |  |  |  |  |  |
| - Yes | 1.08 [0.27; 1.89] | ***<0.011*** | -5.55 [-8.44; -2.65] | ***0.001*** | -0.95 [-2.97; 1.06] | 0.336 | -3.62 [-5.36; -1.87] | ***<0.001*** | -2.32 [-3.84; -0.79] | ***0.005*** |
| **Any CNS Disorder** (ref. No) |  |  |  |  |  |  |  |  |  |  |
| - Yes | 2.20 [0.93; 3.48] | ***0.002*** | -6.95 [-9.95; -3.96] | ***<0.001*** | -3.96 [-7.53; -0.38] | ***0.032*** | -4.12 [-7.20; -1.03] | ***0.011*** | -8.53 [-10.80; -6.25] | ***<0.001*** |
| **Any Musculoskeletal Disorder** (ref. No) |  |  |  |  |  |  |  |  |  |  |
| - Yes | 1.92 [0.95; 2.88] | ***<0.001*** | -6.06 [-8.32; -3.80] | ***<0.001*** | -1.81 [-3.89; 0.26] | 0.084 | -2.46 [-4.63; -0.30] | ***0.028*** | -3.80 [-5.63; -1.98] | ***<0.001*** |
| **Any Respiratory Disorder**  (ref. No) |  |  |  |  |  |  |  |  |  |  |
| - Yes | 1.56 [0.57; 2.54] | ***0.004*** | -4.99 [-7.92; -2.05] | ***0.002*** | -2.04 [-4.76; 0.68] | 0.134 | -0.77 [-3.23; 1.69] | 0.521 | -4.56 [-6.51; -2.61] | ***<0.001*** |
| **Any Gastrointestinal Disorder** (ref. No) |  |  |  |  |  |  |  |  |  |  |
| - Yes | -0.32 [-1.12; 0.48] | 0.410 | 1.27 [-1.81; 4.34] | 0.401 | -1.60 [-4.25; 1.05] | 0.221 | -0.87 [-2.77; 1.04] | 0.353 | -2.22 [-4.49; 0.05] | 0.055 |
| **Any Genitourinary Disorder** (ref. No) |  |  |  |  |  |  |  |  |  |  |
| - Yes | 1.78 [0.42; 3.14] | ***0.013*** | -3.71 [-7.23; -0.19] | ***0.040*** | 3.22 [-1.18; 7.61] | 0.143 | 1.87 [-1.39; 5.12] | 0.246 | -3.43 [-8.06; 1.21] | 0.138 |
| **Any Endocrinological Disorder** (ref. No) |  |  |  |  |  |  |  |  |  |  |
| - Yes | 0.42 [-0.26; 1.10] | 0.209 | -6.04 [-8.50; -3.59] | ***0.000*** | -0.86 [-3.03; 1.30] | 0.416 | 0.57 [-1.85; 2.99] | 0.626 | -3.84 [-6.27; -1.42] | ***0.004*** |
| **Any Cancer Disorder** (ref. No) |  |  |  |  |  |  |  |  |  |  |
| - Yes | 0.21 [-0.71; 1.14] | 0.635 | -6.49 [-11.53; -1.46] | ***0.014*** | 2.89 [0.72; 5.07] | ***0.012*** | 0.82 [-3.39; 5.03] | 0.689 | -7.37 [-12.38; -2.37] | ***0.006*** |
| **Any Dermatological Disorder** (ref. No) |  |  |  |  |  |  |  |  |  |  |
| - Yes | 0.87 [-0.88; 2.63] | 0.313 | -0.10 [-6.20; 5.99] | 0.972 | -1.75 [-6.94; 3.44] | 0.490 | -0.23 [-3.77; 3.31] | 0.892 | -1.30 [-5.89; 3.29] | 0.562 |
| **Any Other Disorder** (ref. No) |  |  |  |  |  |  |  |  |  |  |
| - Yes | 1.37 [0.19; 2.56] | ***0.025*** | -5.88 [-10.12; -1.63] | ***0.009*** | 0.42 [-2.97; 3.81] | 0.799 | -1.48 [-3.50; 0.55] | 0.144 | -2.72 [-7.14; 1.70] | 0,214 |
|  |  |  |  |  |  |  |  |  |  |  |
